# Frailty and neurocognitive impairments in Chinese survivors of childhood cancer

**DOI:** 10.1007/s11764-024-01739-4

**Published:** 2025-01-04

**Authors:** Yihui Wei, Weishang Deng, Kenneth Kin-Wah To, Teddy Tai-Ning Lam, Winnie Wan-Yee Tso, Agnes Sui-Yin Chan, Kirsten K. Ness, Chi Kong Li, Yin Ting Cheung

**Affiliations:** 1 School of Pharmacy, Faculty of Medicine, The Chinese University of Hong Kong, Hong Kong SAR, China; 2 Department of Paediatrics & Adolescent Medicine, The University of Hong Kong, Hong Kong SAR, China; 3 Department of Paediatrics & Adolescent Medicine, Hong Kong Children’s Hospital, Hong Kong SAR, China; 4 Department of Psychology, The Chinese University of Hong Kong, Hong Kong SAR, China; 5 Department of Epidemiology and Cancer Control, St. Jude Children’s Research Hospital, Memphis, TN USA; 6 Department of Paediatrics, Faculty of Medicine, The Chinese University of Hong Kong, Hong Kong SAR, China; 7 Hong Kong Hub of Paediatric Excellence, The Chinese University of Hong Kong, Hong Kong SAR, China

**Keywords:** Childhood cancer, Frailty, Neurocognitive, Survivorship

## Abstract

**Purpose:**

This study aimed to evaluate the prevalence and predictors of frailty and the association between frailty and neurocognitive impairments among Chinese survivors of childhood cancer.

**Methods:**

A total of 185 survivors of childhood cancer were recruited from a long-term follow-up clinic in Hong Kong (response rate: 94.4%; 48.1% female; mean age 28.9 years, standard deviation = 6.7 years). Frailty was assessed using the Fried frailty criteria. Neurocognitive outcomes were evaluated using a performance-based test. Multivariable logistic regression was used to identify the predictors of frailty. Multivariable generalized linear models were used to explore the associations between frailty and cognitive outcomes.

**Results:**

The proportions of survivors with frailty and pre-frailty were 22.7% and 27.0%, respectively. “Frail” survivors were more likely to be diagnosed with cancer at a younger age (odds ratio [OR] = 0.93, 95% confidence interval [CI]: 0.87–0.99, *P* = 0.041) and to have coexisting chronic health conditions (OR = 4.63, 95% CI: 1.68–12.80, *P* = 0.003) than “non-frail” and “pre-frail” survivors. Survivors with frailty exhibited worse attention detectability (unstandardized point estimate [Est] = 4.57, standard error [SE] = 1.69, *P* = 0.007), omissions (Est = 3.68, SE = 1.15, *P* = 0.001), and cognitive flexibility (Est = 8.08, SE = 3.08, *P* = 0.009) than “non-frail” and “pre-frail” survivors.

**Conclusions:**

More than one fifth of the participating Chinese survivors of childhood cancer were identified as phenotypically frail. Frailty was associated with worse performance in attention and executive function.

**Implications for Cancer Survivors:**

The findings highlight the needs for regular monitoring and early interventions that can modify the aging pathway in the cancer continuum, to mitigate frailty and improve psychosocial outcomes during long-term cancer survivorship.

**Supplementary Information:**

The online version contains supplementary material available at 10.1007/s11764-024-01739-4.

## Introduction

Advances in cancer diagnosis and treatment have led to substantial improvements in survival rates among children with cancer in recent years [1]. However, survivors of childhood cancer might have an increased risk of developing late effects of cancer [2]. Long-term follow-up studies have demonstrated that in addition to other chronic morbidities, survivors of childhood cancer experience neurocognitive impairments [3, 4]. According to reports on the St. Jude Lifetime Cohort Study (SJLIFE), the estimated prevalence of severe neurocognitive impairment in long-term survivors of childhood cancer was 14 times higher than that in the general population [5]. In Asian studies, up to 42.8% of survivors of childhood cancer were reported to have neurocognitive impairments [6], which had negative effects on their school performance, employment, productivity, and quality of life [7, 8].

The neurocognitive deficits experienced by cancer survivors can be attributed to the direct effects of cancer itself and the neurotoxic effects of cancer treatment on the brain’s structures and functions [3, 9], and these effects may be further exacerbated by clinical events such as severe acute infection and leukoencephalopathy [10]. However, some recent literature suggests that cancer treatment has indirect effects that lead to long-term neurocognitive impairment. To illustrate, survivors of extracranial cancers who were not exposed to central nervous system (CNS)-related therapy have been reported to have a higher risk of neurocognitive impairments than the general population [11]. Therefore, there is a need to identify the underlying biological mechanism and modifiable risk factors associated with cognitive late effects in cancer survivors.

Increasing evidence in recent years supports the concepts of “accelerated aging” and “premature aging” as mechanisms underlying long-term late effects in cancer survivors [8, 9]. Physiologic frailty, a specific aging phenotype, has been observed in young cancer survivors, and their declining trajectory of neurocognitive functioning has been attributed to accelerated aging [8, 12]. The Childhood Cancer Survivor Study, a large cohort study of survivors of childhood cancer in North America, reported that the prevalence of frailty among adult survivors elevated, being three times higher than that among sibling controls [13]. A low lean muscle level and weakness have also been consistently observed in patients with childhood cancer [14]. From the perspective of biological aging, survivors of childhood cancer were found to have a shorter leukocyte telomere length than their sibling controls, and this phenomenon has been associated with chronic health conditions (CHCs) [15]. Emerging evidence thus suggests that cancer therapy affects intracellular processes, leading to the chronic deterioration of organ function and treatment-related late effects in survivors of childhood cancer.

Normal aging can be altered or accelerated after cancer treatment, resulting in cognitive aging in cancer survivors [16]. Studies have described associations between frailty and impaired cognitive function in cohorts of aging people and breast cancer survivors [17, 18]. A prospective study of the SJLIFE found that among survivors of childhood cancer, those with frailty experienced a greater neurocognitive decline than robust individuals [8]. However, no other published study has validated these findings in other childhood cancer populations. Specifically, though among Chinese community-dwelling older adults, people with frailty exhibit worse performance in cognitive domains than their more robust counterparts and those with pre-frailty [19], findings regarding frailty in Western populations might not directly apply to Asian populations, who have lower levels of skeletal muscle mass and physical capability than Western populations [20]. Lifestyle factors, socioeconomic factors, and genetic predisposition may also affect the susceptibility to aging and cognitive/behavioral outcomes in Western versus Asian survivors of childhood cancer.

Considering the research gap in frailty, the objectives of the current study were (1) to evaluate the prevalence of frailty among Chinese survivors of childhood cancer; (2) to identify the predictors of frailty; and (3) to examine the association between frailty and neurocognitive impairment.

## Methods

### Study design and population

This was an observational, prospective study conducted at the Long-term Follow-up Clinic of the Prince of Wales Hospital in Hong Kong. Survivors of childhood cancer were recruited between January 2022 and October 2023. Ethical approval for the study was obtained from the Joint Chinese University of Hong Kong–New Territories East Cluster Clinical Research Ethics Committee (Ref. no.: 2021.458).

Survivors were eligible for the study if they (1) had been diagnosed with cancer before 18 years of age; (2) were at least 18 years old at the time of recruitment; and (3) had completed treatment for at least 2 years or had survived for at least 5 years since diagnosis. Potential participants were excluded if they (1) had any genetic disorder associated with cognitive impairment (e.g., Down’s syndrome); (2) had a history of non-cancer and/or non-treatment-related conditions known to limit cognitive function (e.g., traumatic brain injury from motor/vehicle accidents); (3) were pregnant or lactating; or (4) had an implanted cardiac device, a contraindication for bioelectrical impedance analysis (BIA). Written informed consent was obtained from all participants.

### Assessment of frailty

Frailty was assessed using the Fried frailty criteria [21], which has previously been used to assess physiologic frailty in survivors of childhood cancer [13, 22]. Participants were defined as pre-frail if they fulfilled two, and frail if they fulfilled three or more, of the following five criteria, as measured using the corresponding assessments: (1) a low lean muscle level [21, 23, 24], measured using BIA; (2) exhaustion [21, 25–27], measured using the Multidimensional Fatigue Scale; (3) low energy expenditure [21, 25, 28, 29], measured using the Chinese University of Hong Kong: Physical Activity Rating for Children and Youth; (4) slowness [21], measured using the 15-feet walking test; and (5) weakness [21, 30], measured by the sitting handgrip strength using a hand-held dynamometer. The specific assessments, definition of the frailty criteria, and sources of reference norms are presented in Supplemental Table 1.

### Neurocognitive outcomes

Neurocognitive outcomes were evaluated using standardized performance-based assessments. The measurement domains were (1) attention, measured using the Continuous Performance Test-III (CPT-III) [31] variables of detectability, commissions, variability, hit reaction time (HRT) block change, and omissions; (2) processing speed, measured using the Grooved Pegboard (GPB) [32] and Trail Making Test (TMT) part A [32]; (3) executive function, measured using CPT perseverations [31] and the TMT part B [32]; and (4) memory, measured using the Modified Taylor Complex Figure (MTCF) [33]. The cognitive outcomes were transformed into age-adjusted *T*-scores (mean = 50; standard deviation [SD] = 10) based on reference norms [34]; this approach has been adopted in other studies on survivors of childhood cancer [25, 35]. All *T*-scores were scaled such that higher scores indicated worse neurocognitive functioning.

### Demographic, clinical, and treatment variables

Sociodemographic information (e.g., age, sex, educational level, employment status, and income level) was collected through both patient interviews and the Clinical Management System (CMS), an electronic data repository of the public healthcare system in Hong Kong. Clinical information (e.g., weight and CHCs), cancer-related information (e.g., cancer diagnosis, age at diagnosis, and time since diagnosis), and treatment-related information (e.g., chemotherapy, radiation, and surgery) were extracted through the CMS. Patients were identified as having received CNS-directed therapies if they had received cranial radiation, neurosurgery, or high-dose methotrexate therapy (> 1,000 mg).

### Statistical analysis

The demographic, socioeconomic, and clinical characteristics of the overall cohort were summarized using descriptive statistics. Frailty phenotypes were identified as both binary (i.e., non-/pre-frail versus frail) and three-level (i.e., non-frail versus pre-frail versus frail) categorical variable. Univariate analysis, including the chi-square test for categorical variables and *t*-test for continuous variables, was used to compare variables between patients grouped by binary frailty phenotypes. Multivariable logistic regression was used to identify the predictors of frailty, which were estimated using odds ratios (ORs) and 95% confidence intervals (CIs). A one-way analysis of variance was used to compare the cognitive outcomes between patients grouped according to three-level frailty phenotypes.

Multivariable generalized linear models were used to explore the associations between frailty and cognitive outcomes while adjusting for age at the time of study, sex, cancer type, CHCs, and CNS-directed therapies. Unstandardized point estimate (Est) and standard error (SE) were used to evaluate the effect sizes of the associations. Subgroup analyses were performed to examine the stratified effects of the associations between frailty and neurocognitive outcomes. All analyses were conducted using SAS 9.4 (SAS Institute, Cary, NC, USA). A two-tailed P value < 0.05 was considered statistically significant.

## Results

### Patient characteristics

Of the 196 survivors who fulfilled the inclusion criteria and were invited to participate in the study, 187 survivors provided informed consent (Supplemental Fig. 1). Of these, 185 survivors have completed the assessments and were included in the analysis (response rate: 94.4%).

The mean age of the participants was 28.9 years (SD = 6.7 years), and 48.1% of the participants were female. The average age at diagnosis was 8.6 years (SD = 5.3 years), and the mean time since diagnosis was 20.3 years (SD = 7.4 years). The cancer diagnoses were hematological cancers (*n* = 118, 63.8%), CNS solid tumors (*n* = 8, 4.3%), and non-CNS solid tumors (*n* = 59, 31.9%). All of the survivors had received chemotherapy. Overall, 18.4% of the survivors had received cranial radiation, 9.2% had received hematopoietic stem cell transplant, and 5.4% had received neurosurgery. The majority of the survivors had CHCs (*n* = 132, 71.3%). The largest proportion of CHCs involved endocrine system diseases (21.6%), followed by cardiovascular (21.1%) and pulmonary organ conditions (9.2%). The demographic and clinical characteristics of the overall study population are presented in Supplemental Table 2.

### Prevalence and predictors of frailty

**Fig. 1:**
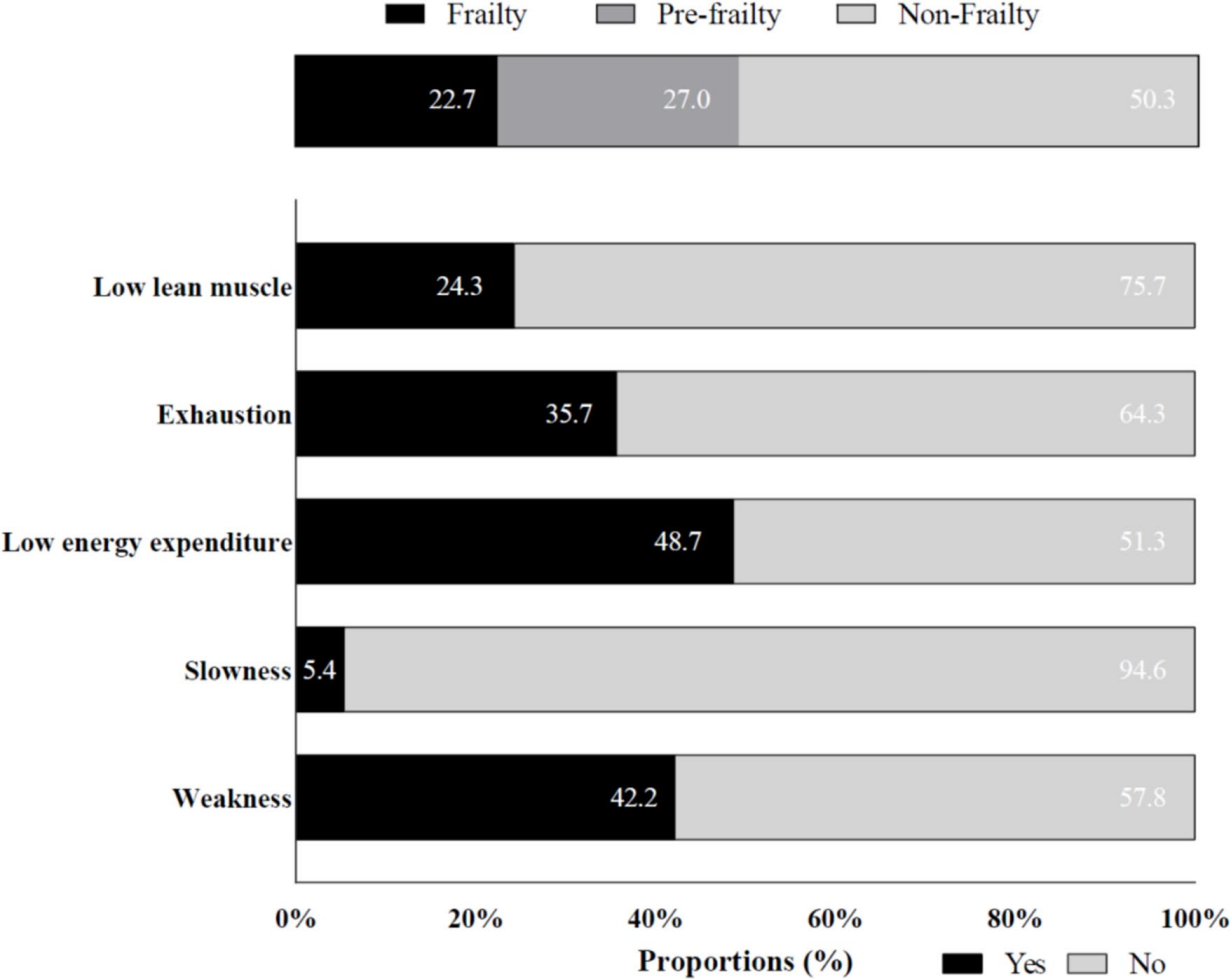
Proportions of Survivors Classified as “Frail” Using the Fried Frailty Criteria. Frailty is assessed using five criteria: low lean muscle, exhaustion, low energy expenditure, slowness and weakness

The proportions of participants with frailty and pre-frailty were 22.7% and 27.0%, respectively (Fig. 1**)**. Nearly half of the survivors presented with low energy expenditure (48.7%) or a weak grip strength (42.2%). Only 5.4% of the survivors were classified as having a “slow walking speed.”

A comparison of the demographic and clinical characteristics of “frail” and “non-/pre-frail” survivors is provided in Table 1. “Frail” survivors were more likely to be unemployed than “non- or pre-frail” survivors (35.7% versus 19.0%, *P* = 0.024), and to have a lower income level (< 20,000 HKD per month, 75.0% versus 47.0%, *P* = 0.003). A higher proportion of “frail” survivors than “non-/pre-frail” survivors had developed CHCs (85.7% versus 67.1%, *P* = 0.019), especially those involving endocrine (33.3% versus 18.2%, *P* = 0.036) and cardiovascular diseases (33.3% versus 17.5%, *P* = 0.027). Age at the time of study, sex, cancer type, and treatment were not significantly different between “frail” and “non-/pre-frail” survivors.

**Table 1:** Demographic and clinical characteristics by frailty phenotype

Characteristics	Non-/pre-frailty	Frailty	*P* value
	***n* (%) / mean (SD)**		
	***n* = 143**	***n* = 42**	
**Age at study (years) mean [SD]**	28.4 [6.6]	28.3 [7.2]	0.954^#^
> 18 – 25	44 (30.8)	13 (31.0)	0.929^*^
≥ 25 – 35	68 (47.5)	21 (50.0)	
≥ 35	31 (21.7)	8 (19.0)	
**Sex**			0.780^*^
Male	75 (52.4)	21 (50.0)	
Female	68 (47.6)	21 (50.0)	
**Education level**			0.439^*^
Below college	72 (50.4)	24 (57.1)	
College and above	71 (49.6)	18 (42.9)	
**Employment status**			0.024^*^
Not employed	27 (19.0)	15 (35.7)	
Employed	115 (81.0)	27 (64.3)	
**Monthly income**			0.003^*^
< 20,000HKD	62 (47.0)	27 (75.0)	
≥ 20,000HKD	70 (53.0)	9 (25.0)	
**Clinical Variables**			
**Cancer type**			0.594^*^
Hematological cancer	92 (64.3)	26 (61.9)	
CNS Solid tumors	5 (3.5)	3 (7.1)	
Non-CNS tumors	46 (32.2)	13 (31.0)	
**Age at diagnosis (years) mean [SD]**	9.0 [5.2]	7.2 [5.4]	0.050^#^
< 5	46 (32.2)	21 (50.0)	0.095^*^
≥ 5 – 10	31 (21.7)	8 (19.1)	
≥ 10 – 18	66 (46.1)	13 (30.9)	
**Time since diagnosis (years) mean [SD]**	19.9 [7.1]	21.7 [8.5]	0.170^#^
**Chronic health conditions**			
No	47 (32.9)	6 (14.3)	0.019^*^
Yes	96 (67.1)	36 (85.7)	
Endocrine	26 (18.2)	14 (33.3)	0.036^*^
Cardiovascular	25 (17.5)	14 (33.3)	0.027^*^
Pulmonary	10 (7.0)	7 (16.7)	0.056^*^
Renal	12 (8.4)	4 (9.5)	0.818^*^
Hepatic	7 (4.9)	3 (7.1)	0.571^*^
**Body mass index (kg/m**^**2**^**) mean [SD]**	22.6 [4.0]	21.1 [4.6]	**0.034**^#^
Underweight	17 (11.9)	13 (31.0)	0.010^*^
Normal	96 (67.1)	24 (57.1)	
Overweight	30 (21.0)	5 (11.9)	
**Treatment-related Variables**			
**Radiation**			0.205^*^
Yes	43 (30.1)	17 (40.5)	
No	100 (69.9)	25 (59.5)	
**Cranial radiation**			0.301^*^
Yes	24 (16.8)	10 (23.8)	
No	119 (83.2)	32 (76.2)	
**Surgery**			0.995^*^
Yes	51 (35.7)	15 (35.7)	
No	92 (64.3)	27 (64.3)	
**Neurosurgery**			0.571^*^
Yes	7 (4.9)	3 (7.1)	
No	136 (95.1)	39 (92.9)	
**HSCT**			0.193^*^
Yes	11 (7.7)	6 (14.3)	
No	132 (92.3)	36 (85.7)	
**Alkylating agent**			0.100^*^
Yes	97 (67.8)	34 (80.9)	
No	46 (32.2)	8 (19.1)	
**Anthracyclines**			0.986^*^
Yes	119 (83.2)	35 (83.3)	
No	24 (16.8)	7 (16.7)	
**Vincristine**			0.572^*^
Yes	92 (64.3)	29 (69.1)	
No	51 (35.7)	13 (30.9)	
**Corticosteroids**			0.917^*^
Yes	83 (58.0)	24 (57.1)	
No	60 (42.0)	18 (42.9)	
**High-dose methotrexate (> 1,000 mg)**			0.464^*^
Yes	35 (24.5)	8 (19.1)	
No	108 (75.5)	34 (80.9)	
**CNS-directed treatment**			0.878^*^
Yes	56 (39.2)	17 (40.5)	
No	87 (60.8)	25 (59.5)	

*SD* standard deviation, *CNS* central nervous system, *HSCT* hematopoietic stem-cell transplantation, CNS-directed treatment included neurosurgery, cranial radiation, or high-dose methotrexate.

*Comparison was conducted using Chi-square test between frailty and non-/pre-frailty group.

#Comparison was conducted using *t*-test between frailty and non-/pre-frailty group.

Boldface indicates statistical significance at *P* < 0.05

The multivariable analysis results (Supplemental Table 3) revealed that survivors who had been diagnosed with cancer at a younger age were more likely to have frailty (OR = 0.93, 95% CI: 0.87–0.99, *P* = 0.041). Survivors with CHCs had 4.63 times higher odds of being frail than those without CHCs (95% CI: 1.68–12.80, *P* = 0.003). Survivors who were employed (OR = 0.43, 95% CI: 0.20–0.92, *P* = 0.030) and had a higher monthly income level (≥ 20,000 HKD) (OR = 0.30, 95% CI: 0.13–0.70, *P* = 0.005) had a lower risk of frailty than those who were unemployed and had lower monthly income level, respectively.

### Associations between frailty and neurocognitive outcomes

**Fig. 2:**
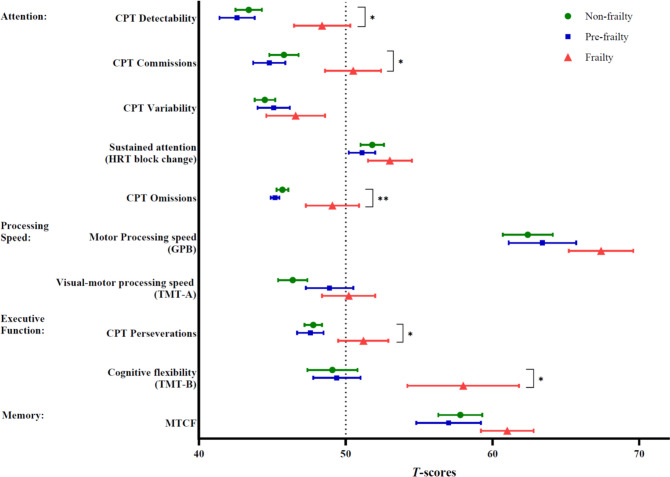
Cognitive Outcomes Grouped by Frailty Phenotype: Non-frailty, Pre-frailty, Frailty. CPT: Continuous Performance Test; HRT: Hit Reaction Time; GPB: Grooved Pegboard; TMT: Trail Making Test; MTCF: Modified Taylor Complex Figure. *T*-scores of cognitive outcomes were displayed as means with standard errors. A higher score was indicative of worse functioning. The vertical line at the score of 50 represents population norm. *Results of multi-level regression; *T*-scores of cognitive outcomes were dependent variables; The frailty phenotype was the independent variable, categorized as frailty, pre-frailty and non-frailty (reference group); Models were adjusted for age at study, sex, cancer type, chronic conditions, and central nervous system-directed treatment (including neurosurgery, cranial radiation, or high-dose methotrexate), with significant differences at *P* < 0.05, **P < 0.01.

Among the three frailty phenotypes, survivors with frailty performed worse in terms of attention measures (CPT detectability, CPT commissions, and CPT omissions) and executive function measures (CPT perseverations and cognitive flexibility) than survivors with pre-frailty and without frailty (Fig. 2).

After adjusting for age, sex, cancer diagnosis, CHCs, and CNS-directed therapies, the multivariable analyses (Table 2) showed that “frail” survivors exhibited more severe impairments in attention and executive function, specifically in terms of CPT detectability (Est = 4.57, SE = 1.69, *P* = 0.007), CPT commissions (Est = 4.14, SE = 1.69, *P* = 0.014), CPT omissions (Est = 3.68, SE = 1.15, *P* = 0.001), CPT perseverations (Est = 3.38, SE = 1.33, *P* = 0.011), and cognitive flexibility (Est = 8.08, SE = 3.08, *P* = 0.009), than “non-frail/pre-frail” survivors. No significant associations were observed between frailty and other neurocognitive outcomes.

**Table 2:** Association between frailty phenotype and cognitive outcomes

Cognitive Outcomes	Frail versus Pre-frail/non-frail		
	Est.*	SE	*P* value
**Attention**			
CPT Detectability	4.57	1.69	**0.007**
CPT Commissions	4.14	1.69	**0.014**
CPT Variability	2.16	1.55	0.161
Sustained attention (CPT HRT Block Change)	1.51	1.40	0.281
CPT Omissions	3.68	1.15	**0.001**
**Processing Speed**			
Motor Processing Speed (GPB)	3.79	2.71	0.162
Visual motor processing speed (TMT-A)	2.08	1.84	0.258
**Executive Function**			
CPT Perseverations	3.38	1.33	**0.011**
Cognitive flexibility (TMT-B)	8.08	3.08	**0.009**
**Memory**			
MTCF	1.92	2.42	0.427

*Est* unstandardized coefficient estimate, *SE* standard error, *CPT* Continuous Performance Test, *HRT* Hit Reaction Time, *GPB* Grooved Pegboard, *TMT* Trail Making Test, *MTCF* Modified Taylor Complex Figure.

*The *T*-scores of cognitive outcomes were the dependent variables, a higher score was indicative of worse functioning; The frailty phenotype was the independent variable, categorized as frail versus non-frail/pre-frail; The models were adjusted for age at study, sex, cancer type, chronic conditions, and central nervous system-directed treatment (including neurosurgery, cranial radiation, or high-dose methotrexate). Boldface indicates significance at *P* < 0.05

The comparison of neurocognitive outcomes between survivors with frailty, survivors with pre-frailty and survivors without frailty in a three-level multivariable analysis (Supplemental Table 4) showed significant differences in attention and executive function only between the non-frail and frail groups. No significant differences were observed in outcomes between the “non-frail” and “pre-frail” groups.

### Results stratified by age at cancer diagnosis

In the analysis stratified by the median age at diagnosis in the overall cohort (8.5 years), significant differences in neurocognitive outcomes between “frail” and “non-frail/pre-frail” survivors were found only among those with a younger age at diagnosis (< 8.5 years). No significant associations were found among those who had been diagnosed with cancer after 8.5 years of age (Supplemental Table 5).

### Results stratified by CNS-directed therapies

Frailty was associated with worse performance on the measures of sustained attention and executive function in the subgroup of survivors who were not exposed to CNS-directed therapies (Supplemental Table 6). No significant association was observed between frailty and executive function in the group of survivors who were previously exposed to CNS-directed therapies.

## Discussion

This is the first study to examine the prevalence of frailty among Asian survivors of childhood cancer. In this study, 22.7% of Chinese young adult survivors of childhood cancer were found to have a frail phenotype. Survivors who had been diagnosed with cancer at a younger age and those with co-existing CHCs had a higher risk of being frail than other survivors. Our results also revealed associations between frailty and impaired attention and executive function, especially among survivors who had been diagnosed with cancer at a younger age or had received non-CNS-directed therapies. Taken together, frailty may be a physiological aging marker of cognitive impairment in survivors of childhood cancer. Collective evidence from the literature may support the development of supportive interventions to slow premature aging and mitigate the adverse impact of aging on neurocognitive outcomes in long-term cancer survivors.

Increasing evidence suggests that survivors of childhood cancer exhibit physiologic frailty as a phenotype of accelerated aging [22]. Although the life expectancy of these survivors is increasing due to advances in treatment, the survivors face a higher risk of becoming frail at an earlier age than the general population [36]. We found that the prevalence of frailty was 22.7% among Chinese survivors of childhood cancer, which is higher than the rates reported in Western cohorts. In a national cohort of Dutch survivors of childhood cancer, the rates of frailty and pre-frailty were 7.4% and 20.3%, respectively [12]. A recent study on the SJLIFE reported that 8.7% of survivors in the cohort were frail and 23.6% were pre-frail [37]. Although frailty has been commonly reported in Asian geriatric populations [38], research focusing on younger cancer survivors remains scarce. More than a third of our cohort exhibited weak handgrip strength, low energy expenditure, and exhaustion, suggesting poor physical functioning and a poor overall health status of the specific population. Differences in frailty rates between Asian and Western populations may exist due to disparities in strength adaptation and body composition than in other ethnic populations [39]. A study also showed that Asian cancer survivors are less likely to engage in physical activity than Caucasian survivors [40]. Consistent with the literature reporting social inequalities in frailty among older adults in Hong Kong [41], we found that survivors whose monthly personal income was lower than the population median (< 20,000 HKD) were more likely to be frail than those who had a higher income. This finding may highlight the need for unique considerations when developing interventions to mitigate frailty in Chinese survivors. Future work should also identify the socio-environmental factors associated with aging phenotypes, so that addressing modifiable risk factors may ameliorate adverse health outcomes in survivors.

Neurocognitive impairments have been shown to negatively impact the functional outcomes of survivors of childhood cancer [4]. Consistent with the findings of the SJLIFE [8], we found a significant association between frailty and neurocognitive impairments in Chinese survivors of childhood cancer, particularly in higher-order thinking processes such as attention and executive function. Importantly, such associations were more apparent in survivors who had not been previously exposed to CNS-directed therapies than in those who had, suggesting that cancer itself and non-neurotoxic treatment contribute to brain function changes through physiologic aging. Healthcare providers should be aware of the need to monitor the frailty status of survivors in high-risk groups and thus provide a more holistic assessment of their physical and health status during long-term survivorship.

The mechanism underlying frailty and cognitive decline after a cancer diagnosis has not been fully illustrated. However, recent studies have reported the associations of accelerated biological aging trajectories between new-onset declines in memory functions in cancer survivors receiving chemotherapy [42, 43]. In particular, cancer and cancer-related treatment were found to have two-way interactions with chronic inflammation and oxidative stress [44]. Inflammation has been recognized as an endogenous factor in aging, leading to organ damage and chronic disease burdens in multiple systems [45]. We also found that survivors who had been diagnosed at an earlier age were more susceptible to frailty, consistent with the literature reporting that younger individuals might be more vulnerable to the damaging effects of cancer itself and related therapies [46]. Future work should focus on understanding the pathways and interactions linking biological processes of aging (e.g., markers of inflammation and oxidative stress, leukocyte telomere length, and epigenetics), frailty, and brain outcomes. These mechanistic studies may guide the development of behavioral interventions that limit cognitive deficits by modifying the aging pathway, as well as the identification of novel pharmaceutical targets for manipulating the mechanisms of cellular aging.

Although the three-level multivariable analysis identified the worst neurocognitive outcomes in frail survivors, we did not find significant differences in functioning between survivors with pre-frailty and without frailty. This result suggests that survivors with pre-frailty could be targeted for monitoring and intervention during the early stages of frailty. Early interventions may mitigate the adverse effects of frailty and aging, and potentially improve the survivors’ neurocognitive function. Studies have shown that dietary and exercise interventions can enhance cognitive function and mitigate frailty in young cancer survivors. For example, micronutrients with antioxidant and anti-inflammatory properties have been shown to reduce the risks of frailty and cognitive decline [47]. In addition, exercise interventions have demonstrated benefits in terms of improved brain volume and structure, muscle mass, and strength [48, 49]. Importantly, local trials in Hong Kong have also demonstrated the effectiveness of adventure-based exercise interventions in reducing cancer-related fatigue and improving grip strength among survivors of childhood cancer [50]. This is promising, as we previously reported that physical activity and health-protective behaviors are associated with improved cognitive function in young adult survivors of sarcoma [51]. These findings may provide a scientific basis upon which to encourage young cancer survivors to adopt multi-component interventions to modify their health outcomes.

Our study has several limitations. First, as this was a cross-sectional study, the interaction or trajectory of frailty and cognitive impairment over time could not be determined. Future longitudinal studies are needed to identify the mechanisms and patterns of the occurrence and development of neurocognitive declines and frailty. Second, the absence of a non-cancer control group limited us to a direct comparison of the prevalence of frailty between cancer survivors and a healthy population. However, extensive evidence in the literature has demonstrated the relationship between cancer and aging phenotypes [52, 53]. Furthermore, the frailty definitions and *T*-scores were generated from normative data with adjustment for age and sex, which to some extent mitigated the concern about not having a healthy control group. The cancer diagnoses of participants in our cohort were heterogenous, and our sample size was probably underpowered for identifying the differential effects of cancer and treatment exposure on frailty status. Future studies involving a young healthy population would enable a more robust assessment of the outcomes and patterns of frailty. Third, we acknowledge that aging is a complex and multifactorial phenomenon. In our study, we adopted only frailty as a phenotype of physiologic aging. The inclusion of other biological markers of aging, such as telomere length or epigenetic age, could facilitate a more comprehensive understanding of aging in the young population. Despite these limitations, our assessment of frailty provides a clinical perspective on physiologic aging and cognitive functions and a foundation for future studies on other aging-related traits in cancer survivors.

## Conclusions

Our study found that more than one fifth of the participating Chinese survivors of childhood cancer were classified as frail based on the Fried frailty criteria. Frailty was associated with worse performance in the domains of attention and executive function, particularly in survivors who had been diagnosed with cancer at a younger age and who had not received CNS-directed therapies. These findings may guide the development of interventions (e.g., exercise and dietary interventions) that can modify the aging pathway early in the cancer continuum. Successful interventions could improve the functional and psychosocial outcomes of survivors before they develop clinical frailty. Future research investigating other biomarkers of aging and the longitudinal trajectory of cognitive function are warranted to elucidate the mechanisms underlying the co-occurrence of aging and functional decline in survivors.

## Supplementary Information

Below is the link to the electronic supplementary material.

Supplementary file1 (DOCX 76 KB)

## Data Availability

The datasets analyzed during the current study are available from the corresponding author on reasonable request.
