# Supplementary material for: Frailty and neurocognitive impairments in Chinese survivors of childhood cancer
Source: J Cancer Surviv. 2025 Jan 4;20(4):1377–87. doi: 10.1007/s11764-024-01739-4 (PMC13375687; doi:10.1007/s11764-024-01739-4)
Supplement: Supplementary file 1 — Supplementary file1 (DOCX 76 KB) [file 11764_2024_1739_MOESM1_ESM.docx]

**Frailty and Neurocognitive Impairments in Chinese Survivors of Childhood Cancer**

*Journal of Cancer Survivorship*

Yihui Wei, MSc^1^, Weishang Deng, MSc^1^, Kenneth Kin-Wah To, PhD^1^, Teddy Tai-Ning Lam, PharmD, PhD^1^, Winnie Wan-Yee Tso, MBBS^2,3^, Agnes Sui-Yin Chan, PhD^4^, Kirsten K Ness, PhD^5^, Chi Kong Li, MBBS, MD^3,6,7^, Yin Ting Cheung, PhD^1,7*^

**Affiliations:**

^1^School of Pharmacy, Faculty of Medicine, The Chinese University of Hong Kong, Hong Kong SAR, China

^2^Department of Paediatrics & Adolescent Medicine, The University of Hong Kong, Hong Kong SAR, China

^3^Department of Paediatrics & Adolescent Medicine, Hong Kong Children’s Hospital, Hong Kong SAR, China

^4^Department of Psychology, The Chinese University of Hong Kong, Hong Kong SAR, China

^5^Department of Epidemiology and Cancer Control, St. Jude Children's Research Hospital, Memphis, TN, USA

^6^Department of Paediatrics, Faculty of Medicine, The Chinese University of Hong Kong, Hong Kong SAR, China

^7^Hong Kong Hub of Paediatric Excellence, The Chinese University of Hong Kong, Hong Kong SAR, China

**Corresponding Author:** Yin Ting Cheung, PhD, Associate Professor, Email address: [yinting.cheung@cuhk.edu.hk](mailto:yinting.cheung@cuhk.edu.hk)

Supplemental Figure 1. Flow Diagram of Recruitment

**Screened**

(n=228)

**Excluded (n=32)**

Non-malignant tumor (n=18)

Developmental or psychiatric disorders before diagnosis of cancer (n=3)

Non-Chinese speaking (n=6)

Currently treated for second cancer or have relapsed (n=5)

**Eligible**

(n=196)

**Non-participants** (n=11)

Defaulted appointment (n=1)

Refusal (did not have time) (n=4)

Refusal (not interested) (n=4)

Withdrawal (did not have time to finish) (n=2)

**Response rate:** 94%

**Completed all assessments**

(n=185)

**Analyzed**

(n=185)

Supplemental Table 1. Assessment of Frailty and Definition of Impairment

|  | Criteria | Method of Evaluation | Definition of Impairment |
| --- | --- | --- | --- |
| 1 | Low lean muscle | Body mass index (BMI);  or lean muscle mass by bioelectrical impedance analysis and anthropometric measurements | Body mass index <18.5kg/m^2^;  or male lean muscle mass ≤7.0kg/m^2^, female lean muscle mass ≤5.7kg/m^2^ [23,24]. |
| 2 | Exhaustion | Paediatric Quality of Life Inventory Multidimensional Fatigue Scale (PedsQL MFS) | Scale total score ≤62.5 (score of 1.3 standard deviations below the population mean of 50) [25-27]. |
| 3 | Low energy expenditure | Chinese University of Hong Kong: Physical Activity Rating for Children and Youth (CUHK-PARCY) | Scale total score <4 [28,29]. |
| 4 | Slowness | 15 feet (4.5 meters) walking test | Test required ≥7 seconds for female <159cm tall or male <173cm; test required ≥6 seconds for female ≥159cm or male≥ 173cm [21]. |
| 5 | Weakness | Sitting hand-grip strength using a hand-held dynamometer | Female grip strength <22.1kg, male grip strength <35.8kg [30]. |

21. Fried LP, Tangen CM, Walston J, et al. Frailty in older adults: evidence for a phenotype. J Gerontol A Biol Sci Med Sci. Mar 2001;56(3):M146-56. doi:10.1093/gerona/56.3.m146

23. Chen W, Jiang H, Yang JX, et al. Body Composition Analysis by Using Bioelectrical Impedance in a Young Healthy Chinese Population: Methodological Considerations. Food Nutr Bull. Jun 2017;38(2):172-181. doi:10.1177/0379572117697534

24. Chen LK, Liu LK, Woo J, et al. Sarcopenia in Asia: consensus report of the Asian Working Group for Sarcopenia. J Am Med Dir Assoc. Feb 2014;15(2):95-101. doi:10.1016/j.jamda.2013.11.025

25. Peng L, Yang LS, Yam P, et al. Neurocognitive and Behavioral Outcomes of Chinese Survivors of Childhood Lymphoblastic Leukemia. *Front Oncol*. 2021;11:655669. doi:10.3389/fonc.2021.655669

26. Varni JW, Burwinkle TM, Katz ER, Meeske K, Dickinson P. The PedsQL in pediatric cancer: reliability and validity of the Pediatric Quality of Life Inventory Generic Core Scales, Multidimensional Fatigue Scale, and Cancer Module. *Cancer*. Apr 1 2002;94(7):2090-106. doi:10.1002/cncr.10428

27. Varni JW, Limbers CA. The PedsQL Multidimensional Fatigue Scale in young adults: feasibility, reliability and validity in a University student population. *Qual Life Res*. Feb 2008;17(1):105-14. doi:10.1007/s11136-007-9282-5

28. Kong AP, Choi KC, Li AM, et al. Association between physical activity and cardiovascular risk in Chinese youth independent of age and pubertal stage. *BMC Public Health*. Jun 3 2010;10:303. doi:10.1186/1471-2458-10-303

29. Chung OK, Li HC, Chiu SY, Ho KY, Lopez V. The impact of cancer and its treatment on physical activity levels and behavior in Hong Kong Chinese childhood cancer survivors. *Cancer Nurs*. May-Jun 2014;37(3):E43-51. doi:10.1097/NCC.0b013e3182980255

30. Yu R, Ong S, Cheung O, Leung J, Woo J. Reference Values of Grip Strength, Prevalence of Low Grip Strength, and Factors Affecting Grip Strength Values in Chinese Adults. *J Am Med Dir Assoc*. Jun 1 2017;18(6):551.e9-551.e16. doi:10.1016/j.jamda.2017.03.006

Supplemental Table 2. Demographic and Clinical Characteristics of the Study Population

| **Characteristics** | **N (%) / Mean (SD)** |
| --- | --- |
| **Age at study (years) mean [SD]** | 28.9 [6.7] |
| >18 – 25 | 57 (30.8) |
| ≥25 – 35 | 89 (48.1) |
| ≥35 | 39 (21.1) |
| **Sex** |  |
| Male | 96 (51.9) |
| Female | 89 (48.1) |
| **Education** |  |
| Below college | 96 (51.9) |
| College and above | 89 (48.1) |
| **Employment status** |  |
| Employed | 142 (77.2) |
| Not employed | 42 (22.8) |
| **Monthly income** |  |
| <20,000HKD | 89 (53.0) |
| ≥20,000HKD | 79 (47.0) |
|  |  |
| **Clinical Variables** |  |
| **Cancer type** |  |
| Hematological cancers | 118 (63.8) |
| CNS Solid tumors | 8 (4.3) |
| Non-CNS solid tumors | 59 (31.9) |
| **Age at diagnosis (years) mean [SD]** | 8.6 [5.3] |
| <5 | 67 (36.2) |
| ≥5 – 10 | 39 (21.1) |
| ≥10 – 18 | 79 (42.7) |
| **Time since diagnosis (years) mean [SD]** | 20.3 [7.4] |
| **Chronic conditions** |  |
| No | 53 (28.7) |
| Yes | 132 (71.3) |
| Endocrine | 40 (21.6) |
| Cardiovascular | 39 (21.1) |
| Pulmonary | 17 (9.2) |
| Renal | 16 (8.7) |
| Hepatic | 10 (5.4) |
| **Body mass index (kg/m^2^) mean [SD]** | 22.3 [4.2] |
| Underweight | 30 (16.2) |
| Normal | 120 (64.9) |
| Overweight | 35 (18.9) |
|  |  |
| **Treatment-related Variables** |  |
| **Radiation** |  |
| Yes | 60 (32.4) |
| No | 125 (67.6) |
| **Cranial radiation** |  |
| Yes | 34 (18.4) |
| No | 151 (81.6) |
| **Surgery** |  |
| Yes | 66 (35.7) |
| No | 119 (64.3) |
| **Neurosurgery** |  |
| Yes | 10 (5.4) |
| No | 175 (94.6) |
| **HSCT** |  |
| Yes | 17 (9.2) |
| No | 168 (90.8) |
| **Chemotherapy** |  |
| Yes | 185 (100) |
| No | 0 |
| **Alkylating agent** |  |
| Yes | 131 (70.8) |
| No | 54 (29.2) |
| **Anthracyclines** |  |
| Yes | 154 (83.2) |
| No | 31 (16.8) |
| **Vincristine** |  |
| Yes | 121 (65.4) |
| No | 64 (34.6) |
| **Corticosteroids** |  |
| Yes | 107 (57.8) |
| No | 78 (42.2) |
| **High-dose methotrexate (>1,000mg)** |  |
| Yes | 43 (23.2) |
| No | 142 (76.8) |
| **CNS-directed treatment** |  |
| Yes | 73 (39.5) |
| No | 112 (60.5) |

SD: standard deviation; CNS: central nervous system; HSCT: hematopoietic stem-cell transplantation; CNS-directed treatment included neurosurgery, cranial radiation, or high-dose methotrexate.

Supplemental Table 3. Association between Frailty Phenotype and Patient Characteristics

| **Independent Variables** | **aOR of Frailty*** | **95% CI** | **P value** |
| --- | --- | --- | --- |
| **Age at study^#^** | 0.99 | 0.95-1.05 | 0.921 |
| **Age at diagnosis** | 0.93 | 0.87-0.99 | **0.041** |
| **Sex** |  |  |  |
| Male | Ref |  |  |
| Female | 1.08 | 0.53-2.17 | 0.839 |
| **Cancer type** |  |  |  |
| Hematological cancer | Ref |  |  |
| CNS Solid tumors | 2.67 | 0.57-12.5 | 0.213 |
| Non-CNS tumors | 1.13 | 0.52-2.45 | 0.762 |
| **Education** |  |  |  |
| Below college | Ref |  |  |
| College and above | 0.77 | 0.38-1.58 | 0.479 |
| **Employment** |  |  |  |
| Unemployed | Ref |  |  |
| Employed | 0.43 | 0.20-0.92 | **0.030** |
| **Monthly income** |  |  |  |
| < 20,000HKD | Ref |  |  |
| ≥ 20,000HKD | 0.30 | 0.13-0.70 | **0.005** |
| **Relapse** |  |  |  |
| No | Ref |  |  |
| Yes | 1.84 | 0.72-4.68 | 0.204 |
| **Chronic health conditions** |  |  |  |
| No | Ref |  |  |
| Yes | 4.63 | 1.68-12.8 | **0.003** |
| **Radiation** |  |  |  |
| No | Ref |  |  |
| Yes | 1.54 | 0.74-3.20 | 0.247 |
| **Surgery** |  |  |  |
| No | Ref |  |  |
| Yes | 1.22 | 0.31-4.87 | 0.776 |
| **HSCT** |  |  |  |
| No | Ref |  |  |
| Yes | 2.14 | 0.72-6.35 | 0.172 |
| **CNS-directed treatment** |  |  |  |
| No | Ref |  |  |
| Yes | 1.14 | 0.56-2.33 | 0.725 |

aOR: adjusted odds ratio; CI: confidence interval; CNS: central nervous system; HSCT: hematopoietic stem-cell transplantation; CNS-directed treatment included neurosurgery, cranial radiation, or high-dose methotrexate.

*The frailty phenotype was the dependent variable, categorized as frailty versus non-/pre-frailty; patient characteristics were defined as independent variables in the multivariable regression. The models were adjusted for age at diagnosis, sex and cancer type.

^#^“Age at diagnosis” was not adjusted in the model with “age at study” as the independent variable due to multicollinearity concerns.

Boldface indicated statistical significance at P<0.05.

Supplemental Table 4. Association between Frailty Phenotype and Cognitive Outcomes Evaluated using Multi-level Regression Model

| **Cognitive outcomes** | **Level** | **Est.*** | **SE** | **P value** |
| --- | --- | --- | --- | --- |
| **Attention** | | | | |
| CPT Detectability | Non-frailty | Ref |  |  |
|  | Pre-frailty | -0.76 | 1.72 | 0.660 |
|  | Frailty | 4.28 | 1.82 | **0.019** |
| CPT Commissions | Non-frailty | Ref |  |  |
|  | Pre-frailty | -1.04 | 1.72 | 0.545 |
|  | Frailty | 3.74 | 1.82 | **0.040** |
| CPT Variability | Non-frailty | Ref |  |  |
|  | Pre-frailty | 0.76 | 1.57 | 0.628 |
|  | Frailty | 2.46 | 1.66 | 0.139 |
| Sustained attention (CPT HRT Block Change) | Non-frailty | Ref |  |  |
|  | Pre-frailty | -0.55 | 1.42 | 0.698 |
|  | Frailty | 1.30 | 1.50 | 0.389 |
| CPT Omissions | Non-frailty | Ref |  |  |
|  | Pre-frailty | -0.33 | 1.17 | 0.780 |
|  | Frailty | 3.56 | 1.23 | **0.004** |
| **Processing Speed** | | | | |
| Motor Processing Speed (GPB) | Non-frailty | Ref |  |  |
|  | Pre-frailty | -0.59 | 2.80 | 0.832 |
|  | Frailty | 3.56 | 2.92 | 0.223 |
| Visual motor processing speed (TMT-A) | Non-frailty | Ref |  |  |
|  | Pre-frailty | 0.78 | 1.9 | 0.683 |
|  | Frailty | 2.38 | 1.99 | 0.231 |
| **Executive Function** | | | | |
| CPT Perseverations | Non-frailty | Ref |  |  |
|  | Pre-frailty | -0.33 | 1.36 | 0.809 |
|  | Frailty | 3.25 | 1.43 | **0.023** |
| Cognitive flexibility (TMT-B) | Non-frailty | Ref |  |  |
|  | Pre-frailty | -0.16 | 3.18 | 0.961 |
|  | Frailty | 8.02 | 3.33 | **0.016** |
| **Memory** | | | | |
| MTCF | Non-frailty | Ref |  |  |
|  | Pre-frailty | -1.19 | 2.46 | 0.628 |
|  | Frailty | 1.47 | 2.60 | 0.571 |

Est: unstandardized coefficient estimate; SE: standard error; CPT: Continuous Performance Test; HRT: Hit Reaction Time; GPB: Grooved Pegboard; TMT: Trail Making Test; MTCF: Modified Taylor Complex Figure.

*The *T*-scores of cognitive outcomes were the dependent variables, a higher score was indicative of worse functioning; The frailty phenotype was the independent variable, categorized as frailty versus pre-frailty versus non-frailty; The models were adjusted for age at study, sex, cancer type, chronic conditions, and central nervous system-directed treatment (including neurosurgery, cranial radiation, or high-dose methotrexate). Boldface indicates significance at P<0.05.

Supplemental Table 5. Association between Frailty Phenotype and Cognitive Outcomes Stratified by Age at Diagnosis

|  | **Survivors with Frailty versus Survivors with Pre-frailty & without Frailty** | | | | | |
| --- | --- | --- | --- | --- | --- | --- |
|  | **Cancer diagnosis age <8.5 years***  **n=92** | | | **Cancer diagnosis age ≥8.5 years**  **n=93** | | |
| **Cognitive Outcomes** | **Est.^#^** | **SE** | **P value** | **Est.** | **SE** | **P value** |
| **Attention** |  |  |  |  |  |  |
| CPT Detectability | 6.00 | 2.44 | **0.015** | -0.34 | 2.43 | 0.889 |
| CPT Commissions | 5.45 | 2.47 | **0.028** | -1.18 | 2.35 | 0.614 |
| CPT Variability | 4.13 | 2.48 | 0.097 | -1.03 | 1.84 | 0.576 |
| Sustained attention (CPT HRT Block Change) | 2.35 | 1.93 | 0.224 | 0.58 | 2.25 | 0.797 |
| CPT Omissions | 5.67 | 2.13 | **0.008** | 0.31 | 0.68 | 0.648 |
| **Processing Speed** |  |  |  |  |  |  |
| Motor Processing Speed (GPB) | 3.19 | 4.22 | 0.450 | 0.86 | 3.58 | 0.810 |
| Visual motor processing speed (TMT-A) | 3.29 | 2.26 | 0.145 | 1.36 | 3.16 | 0.659 |
| **Executive Function** |  |  |  |  |  |  |
| CPT Perseverations | 4.93 | 2.23 | **0.027** | 0.06 | 1.54 | 0.967 |
| Cognitive flexibility (TMT-B) | 11.80 | 4.74 | **0.013** | 1.66 | 4.21 | 0.693 |
| **Memory** |  |  |  |  |  |  |
| MTCF | 2.26 | 3.71 | 0.541 | 0.32 | 3.57 | 0.929 |

Est: unstandardized coefficient estimate; SE: standard error; CPT: Continuous Performance Test; HRT: Hit Reaction Time; GPB: Grooved Pegboard; TMT: Trail Making Test; MTCF: Modified Taylor Complex Figure.

*The median age at cancer diagnosis of the cohort was 8.5 years old.

#The *T*-scores of cognitive outcomes were the dependent variables, a higher score was indicative of worse functioning; The frailty phenotype was the independent variable, categorized as frail versus non-frail/pre-frail; The models were adjusted for age at study, sex, cancer type, chronic health conditions, and central nervous system-directed treatment (including neurosurgery, cranial radiation, or high-dose methotrexate). Boldface indicates significance at P<0.05.

Supplemental Table 6. Association between Frailty Phenotype and Cognitive Outcomes Stratified by CNS-directed Treatment

|  | **Survivors with Frailty versus Survivors with Pre-frailty & without Frailty** | | | | | |
| --- | --- | --- | --- | --- | --- | --- |
|  | **Not received CNS-directed treatment**  **n=112** | | | **Received CNS-directed treatment**  **n=73** | | |
| **Cognitive Outcomes** | **Est.*** | **SE** | **P value** | **Est.** | **SE** | **P value** |
| **Attention** |  |  |  |  |  |  |
| CPT Detectability | 4.16 | 2.33 | 0.074 | 4.73 | 2.36 | **0.045** |
| CPT Commissions | 3.65 | 2.27 | 0.108 | 4.45 | 2.54 | 0.079 |
| CPT Variability | 3.53 | 2.29 | 0.124 | -0.01 | 1.61 | 0.997 |
| Sustained attention (CPT HRT Block Change) | 3.17 | 1.53 | **0.038** | -0.88 | 2.71 | 0.744 |
| CPT Omissions | 5.11 | 1.82 | **0.005** | 1.54 | 0.76 | **0.043** |
| **Processing Speed** |  |  |  |  |  |  |
| Motor Processing Speed (GPB) | 3.58 | 3.78 | 0.344 | 4.14 | 3.85 | 0.282 |
| Visual motor processing speed (TMT-A) | 2 | 2.14 | 0.348 | 2.59 | 3.33 | 0.437 |
| **Executive Function** |  |  |  |  |  |  |
| CPT Perseverations | 3.98 | 2.02 | **0.049** | 2.12 | 1.32 | 0.109 |
| Cognitive flexibility (TMT-B) | 6.43 | 3.03 | **0.034** | 10.4 | 6.29 | 0.100 |
| **Memory** |  |  |  |  |  |  |
| MTCF | 1.14 | 3.14 | 0.716 | 2.97 | 3.93 | 0.449 |

CNS: central nervous system; Est: unstandardized coefficient estimate; SE: standard error; CPT: Continuous Performance Test; HRT: Hit Reaction Time; GPB: Grooved Pegboard; TMT: Trail Making Test; MTCF: Modified Taylor Complex Figure.

*The *T*-scores of cognitive outcomes were the dependent variables, a higher score was indicative of worse functioning; The frailty phenotype was the independent variable, categorized as frailty versus non-/pre-frailty; The models were adjusted for age at study, sex, cancer type, and chronic health conditions. CNS-directed treatment included cranial radiation, neurosurgery or high-dose methotrexate. Boldface indicates significance at P<0.05.
